# A Decade of Complex Regional Pain Syndrome Following Orthopedic Wrist Surgery: A Case Report and a Literature Review

**DOI:** 10.7759/cureus.42540

**Published:** 2023-07-27

**Authors:** Kateryna Georgiyeva, Harendra Kumar, Vania E Fernandez

**Affiliations:** 1 Internal Medicine, Memorial Healthcare System, Pembroke Pines, USA; 2 Medicine and Surgery, Dow University of Health Sciences, Karachi, PAK; 3 Pain Management, Broward Spine Institute, Hollywood, USA

**Keywords:** chronic steroids, opioids in chronic non cancer pain, ketamine infusion, neuropathic pain treatment, stellate ganglion blockade, epidural injections, regional nerve block, dupuytren contracture, orthopedic hand surgery, complex regional pain

## Abstract

Every day, people of all ages in the United States break bones, with traumatic injury being the most common way, and wrist injury being in the top five most common areas in which bones break. Traumatic fractures are managed with either surgical or nonsurgical approaches. The surgical approach utilizes ortho procedures such as internal fixation and reduction, while the nonsurgical approach consists of procedures like RICE, ace bandage, and so on. However, in rare cases, patients are left with a peculiar constellation of symptoms, which cause edema, pain, skin changes, and loss of function at the trauma site. This occurrence is termed complex regional pain syndrome. Here, we present the case of a 55-year-old female patient who suffered a traumatic wrist injury. The trauma was fixed by pinning ORIF orthopedic surgery, and the patient developed manifestations of complex regional pain syndrome around 10 days postoperatively. In this case report, we describe the variation and complexity of symptoms in the patient over the course of a decade after the original injury. The case report explains the pain management therapies that reduced the patient's symptoms and highlights the ones that were ineffective. We have included some less frequently used yet effective treatments and shed light on how this disease affected the patient’s overall well-being.

## Introduction

Complex regional pain syndrome (CRPS) is a complicated and often devastating syndrome characterized by persistent and excruciating pain in the extremity after an injury or surgical treatment [1]. The purpose of this case report and literature analysis is to emphasize the entire presentation of CRPS in Ms. M, a 55-year-old female patient, following pinning ORIF wrist surgery by orthopedic surgery for a serious displaced wrist fracture. The report provides an in-depth analysis of her clinical presentation, the progression of her symptoms through time, and the various therapeutic modalities used to manage her condition.

CRPS is a complex neurological condition characterized by peripheral and central nervous system malfunctions, resulting in a range of sensory, motor, and autonomic problems [1]. Acute discomfort, edema, skin abnormalities, and a decreased range of motion in the affected limb are frequent symptoms of the condition [1,2]. The patient's initial complaint of right extremity swelling, excruciating pain, and skin changes in the right wrist highlighted the development of CRPS after orthopedic wrist surgery. She also had neurologic dysfunction, which included dysesthesia, hyperesthesia, and decreased motor function. Ms. M's subsequent illness history indicates the progressive nature of CRPS, as she developed additional symptoms and experienced its spread to other body parts [2]. She developed symptoms such as bilateral extremity edema, stiffness, spasticity, and soreness in the upper and lower limbs over time. 

The treatment of CRPS necessitates a multifaceted approach that combines several therapeutic modalities [3]. Physical therapy, pharmacological treatments such as nonsteroidal anti-inflammatory drugs (NSAIDs), opioids, and adjuvant medications, as well as interventional pain procedures such as stellate ganglion blocks, lumbar sympathetic nerve blocks, and other nerve blocks were used in Ms. M's case. Furthermore, throughout treatment, ketamine in IV infusions and oral tablet form was utilized, as it is known for its ability to reduce both pain and concomitant depressive symptoms, such as anxiety, depression, and apathy.

The psychological impact of CRPS cannot be overlooked, as patients often experience considerable emotional distress and functioning problems as a result of prolonged pain [4]. Ms. M's journey demonstrates the significant effect CRPS may have on an individual's well-being, with the progression of her condition resulting in depression and the need for assistive devices such as a cane.

Moreover, our case report and literature review shed light on the significant development of CRPS after orthopedic wrist surgery. The complexities of Ms. M's clinical presentation, the rising severity of her symptoms, and the multidisciplinary approach to her treatment highlight the difficulties in recognizing, treating, and addressing the psychological consequences of CRPS. Understanding such conditions is critical for furthering clinical knowledge, refining treatment strategies, and improving outcomes for people suffering from this dreadful disease.

## Case presentation

Ms. M, a 55-year-old female patient with a past medical history of hypothyroidism and depression, and allergy to hydrocodone-acetaminophen first presented to our pain management clinic 10 years prior after undergoing orthopedic surgery for a traumatic wrist fracture. At the time of the operation, one plate and four screws were inserted into the patient’s wrist by an orthopedic surgeon, and concomitant carpal tunnel surgery was performed. She was referred to our pain management clinic by the same orthopedic surgeon for treatment of refractory wrist and right upper extremity pain following the surgery, approximately 10 days postoperatively. The patient’s presenting complaints were significant right extremity edema, 8/10 burning pain in the area of the operation, as well as skin changes in the same area, including redness and mottling. There was evidence of neurologic dysfunction in the form of dysesthesia, allodynia, and hyperesthesia. She was unable to produce a gripping motion (make a fist) using the right hand, and experienced limited range of motion and pain in the wrist and right upper extremity. Taking all these findings into consideration, a diagnosis of CRPS was made at our clinic.

**Figure 1:**
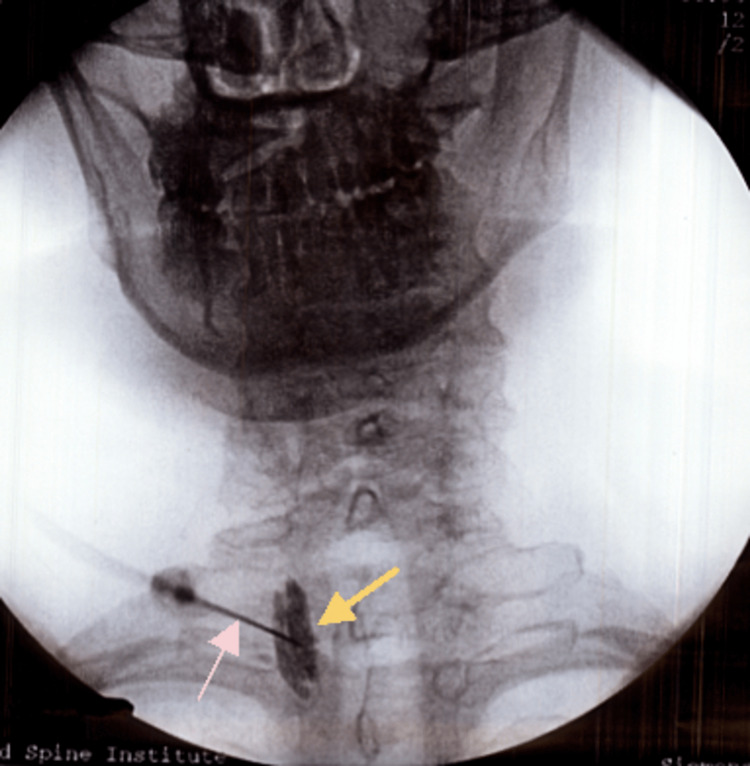
Right-sided stellate ganglion block under fluoroscopic guidance using C-arm. The image shows the site of contrast and medication injection (yellow arrow) and the catheter used to guide injection (pink arrow).

A multimodal treatment regimen was initiated, which included physical therapy, transdermal fentanyl 25 micrograms/hour for baseline pain control, naproxen 500 mg bid and 5/325 mg oxycodone-acetaminophen for breakthrough pain, and lidocaine patches for localized pain. Alongside the prescribed therapy, a series of three stellate ganglion blocks were done, utilizing lidocaine and methylprednisolone. This intervention led to a 50% improvement in pain, as per the patient. However, stiffness and spasticity were still present in the right upper extremity. Figure 1 shows a right-sided stellate ganglion block; the block was performed under fluoroscopic guidance with the use of contrast, and the image was taken with the help of a C-arm.

**Figure 2:**
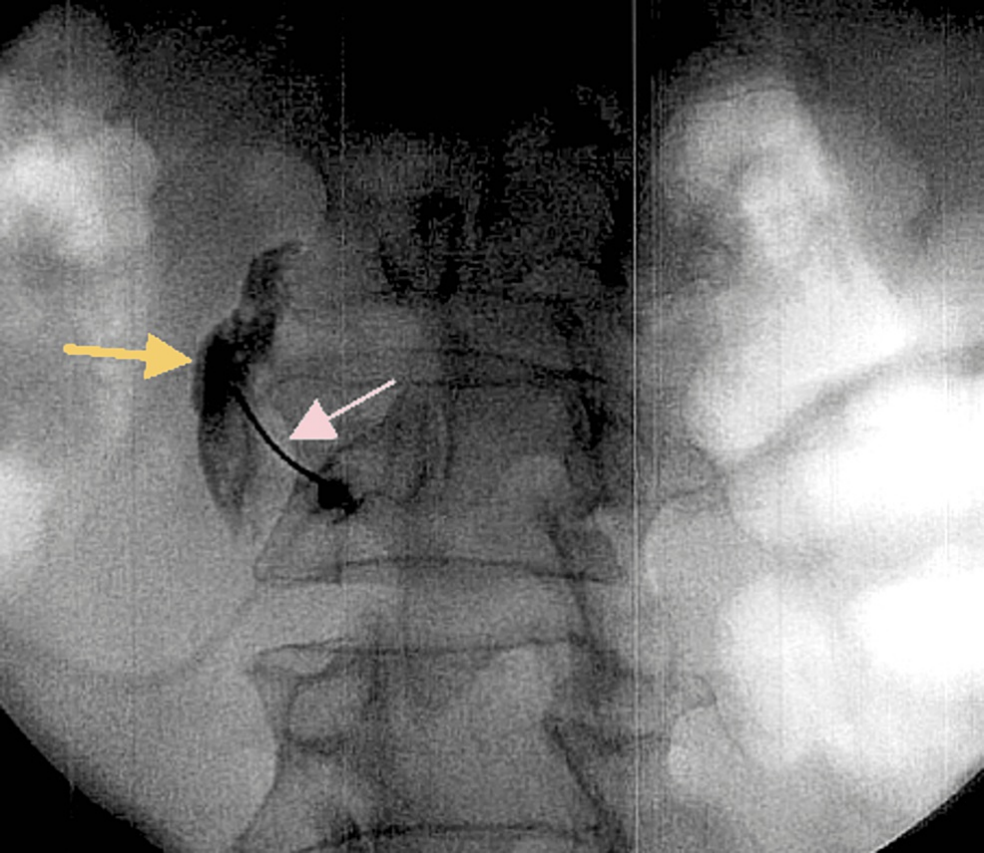
Left L5-S1 lumbar sympathetic nerve block in anteroposterior view. The image shows the site of contrast and medication injection (yellow arrow) and the catheter used to guide injection (pink arrow).

**Figure 3:**
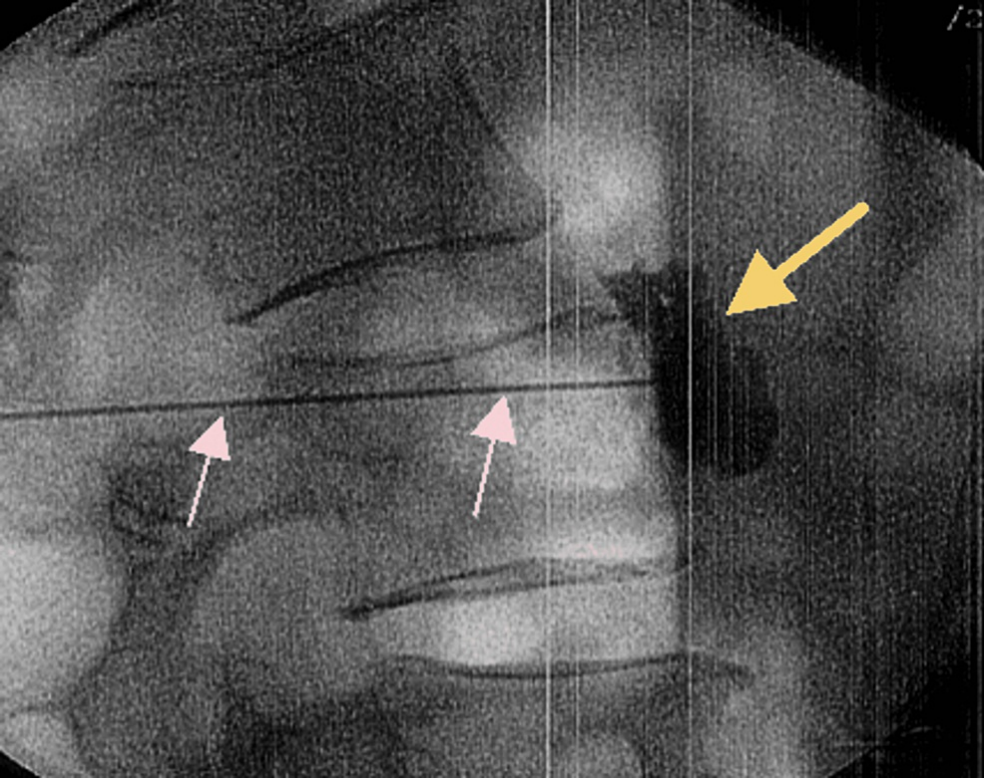
Left L5-S1 lumbar sympathetic nerve block in lateral view. The image shows the site of contrast and medication injection (yellow arrow) and the catheter used to guide injection (pink arrows).

Physical therapy and the medication regimen led to an improvement in the patient’s pain over the course of a couple of months, and she noted moderate improvement in the upper extremity stiffness and burning. However, at a follow-up visit at the pain management clinic, the patient complained of new-onset lumbar pain and bilateral lower extremity pain and edema. She denied any trauma to the area. Physical examination was positive for lumbar paraspinal muscle spasm, exacerbated by spinal flexion, and a positive straight leg raise test. This led to a referral for a lower extremity ultrasound as part of a vascular workup. The study yielded no specific findings and determined the cause of leg pain to be vascular vs. neuropathic pain. Following this, an MRI study of the lumbar spine was done, which showed multiple disc bulges, with mild facet joint spondylolysis, pointing to a neuropathic cause of symptoms. The patient’s bilateral lower extremity pain continued, and new symptoms of edema and burning pain were noted in the legs, knees, feet, and ankles at a follow-up visit in two months. A lumbar epidural at the L5-S1 spinal level was performed, which partially alleviated the lower extremity pain. Figures 2, 3 show a left lumbar sympathetic nerve block that was performed under fluoroscopic guidance with the use of contrast, and the images were taken using a C-arm in anteroposterior and lateral views, respectively.

Two years after the original diagnosis, Ms. M reported new-onset CRPS symptoms in the left upper extremity, indicating further appearance of symptoms contralateral to the original right upper extremity side of the injury. Bilateral upper extremities showed signs of increasing muscle atrophy, weakness, swelling, and joint stiffness. Further stellate ganglion blocks for both right and left upper extremity pain were performed over the course of years, with a wise recommendation from the treating pain management physician to perform them without a steroid component. This change allowed local preservation of bone at the injection site, while still achieving pain relief. While these interventions helped control CRPS pain, Ms. M developed new-onset changes in the color of the skin in the upper extremities, specifically the appearance of vertical lines on the palmar surface of the fingers in the right hand, as well as bilateral reproduction of pain on range-of-motion testing of the upper extremities.

At the three-year mark, new ways of pain relief were discussed with Ms. M. The patient inquired about spinal cord stimulation therapy and intrathecal pain pump insertion for more vigorous pain relief. A beneficial new addition to Ms. M’s multimodal pain regimen included IV ketamine. At this moment in time, Ms. M’s medication regimen included the use of transdermal and short-acting opioids for pain control daily. She experienced adverse effects from chronic opioid use, including constipation, which led to a left inguinal hernia requiring surgical repair, dizziness, and nausea. Of note, the hernia repair surgery left Ms. M with devastating pain at the site of surgery, as this is not uncommon in patients suffering from CRPS. Further, the spread of CRPS symptoms was also noted. Ms. M acquired a cane for ambulation due to the instability in the legs and knees from CRPS, which was described as a sensation of “buckling knees.” Intermittent swelling and bilateral lower extremity pain improved greatly with multiple lumbar sympathetic nerve blocks done at our clinic. 

At the four-five-year mark after the diagnosis of her illness, the patient noted the increasing need for interventional pain management techniques like nerve blocks to control her CRPS pain with the same effectiveness as before. The patient mentioned that these nerve blocks allowed her to perform activities of daily living, and without these treatments, the CRPS pain would not allow her to leave her home. A trial of sphenopalatine ganglion nerve block was recommended at this time for new-onset throbbing head and neck pain. The existing pain in the bilateral lower extremity worsened and the patient also developed new-onset right hip pain, which was later diagnosed as right hip arthritis. This pain was treated with a diagnostic block of the articular branches of the obturator and femoral nerves of the hip joint, which proved to be diagnostic. Definite pain relief in the right hip joint was achieved with radiofrequency ablation to the same area. The patient was also found to have right trochanteric bursitis, and this pain was treated with a methylprednisolone injection into the bursa. The patient additionally noted trigger finger symptoms in the fourth digit of the left hand, which responded well to a steroid trigger point injection and improved range of motion. 

After six-seven years of dealing with CRPS, Ms. M expressed complaints of left trochanteric bursa pain with radiation to the left thigh and iliotibial band. Along with already present right trochanteric bursitis symptoms, this new-onset pain indicated a bilateral spread of bursitis symptoms. Another concern included weight loss and further muscle atrophy in the upper extremities from worsening CRPS. Ms. M also had new-onset trigger finger symptoms in the fourth digit of the right hand, indicating bilateral spread. 

By the eight-nine-year mark, the patient continued to struggle with CRPS symptoms in all four extremities. Improvement of overall pain was noted with the addition of 400 mg of oral ketamine, both greatly improving mood and alleviating pain. An adjunct therapy that was added included once-weekly acupuncture. The patient stated that significant relief was felt by utilizing this technique to treat her CRPS pain. A physical therapy regimen, continued stretching exercises, and adding regular physical exercise continued to help with mobility. These changes allowed the patient to decrease the dose of daily opioid medications transdermal base fentanyl to 50 micrograms, along with an addition of 800 mg of acetaminophen and 5 mg of a muscle relaxant, tizanidine, to achieve the same rate of pain control as before. A new-onset Dupuytren's contracture was noted at this time, which was treated with collagenase *Clostridium histolyticum* enzymatic therapy by an orthopedic specialist.

At this time, Ms. M continues to live with CRPS almost a decade after the original diagnosis. However, with proper tailoring of her medication regimen, physical therapy, acupuncture, exercise, stretching routines, and regular visits to our interventional pain management clinic and psychologist, she is thriving despite the many challenges posed by CRPS. Additionally, she currently does not require the use of an assistive device for ambulation.

## Discussion

CRPS is a multifaceted disorder characterized by substantial and usually exaggerated pain, sensory abnormalities, and autonomic dysfunction [1]. It may develop as a result of a variety of factors, including trauma, surgery, or even minor injuries. This report highlights the case of Ms. M, a 55-year-old female patient, who had substantial CRPS after orthopedic wrist surgery. The clinical characteristics, diagnostic issues, and treatment techniques for severe CRPS in the context of orthopedic wrist surgery will be discussed in detail.

CRPS is diagnosed based on the clinical findings obtained from the patient's medical history and physical examination. The International Association for the Study of Pain (IASP) developed diagnostic criteria, including the Orlando Criteria for Complex Regional Pain Syndrome and the Budapest Clinical Diagnostic Criteria for Complex Regional Pain Syndrome [1]. CRPS is classified into two types: type I and type II, which differ based on the presence or absence of measurable nerve injury. CRPS type I often develops after a noxious episode, is not limited to a specific peripheral nerve distribution, and is disproportionate to the initial triggering event. Symptoms include edema, changes in cutaneous blood flow, abnormal pseudomotor activity, allodynia, and hyperalgesia. The illness typically affects the affected extremity's distal area or exhibits a distal-to-proximal gradient. CRPS type II, on the other hand, is characterized by searing pain, hypersensitivity to touch (allodynia), and exaggerated reaction to any stimuli (hyperpathia) in a specific location of the limb after partial damage to a nerve or one of its primary branches that innervates that region [1,2]. In the case of our patient, definite damage to the wrist nerves from orthopedic trauma is the known injury that precipitated the development of CRPS type I.

Ms. M's clinical picture was indicative of advanced CRPS with significant symptoms and functional limitations. Edema, searing pain, skin abnormalities, and neurologic dysfunction in the form of dysesthesia and hyperesthesia were all described by the patient. She also had impaired hand function and a limited range of motion in the whole right wrist and right upper extremities. These symptoms, when combined, pointed to the development of widespread CRPS, emphasizing the severity of the condition and its impact on Ms. M's daily life. Because of overlapping symptoms with postoperative disorders such as delirium, wound infection, and seroma and a lack of specialized diagnostic testing, diagnosing substantial CRPS following orthopedic wrist surgery may be difficult. However, a proper diagnosis requires a thorough evaluation of the patient's history and clinical presentation and the exclusion of other possible causes, such as fibromyalgia and other rheumatologic conditions. Ms. M's diagnosis of extensive CRPS was supported by the presence of characteristic symptoms such as excruciating pain, skin abnormalities, and neurologic dysfunction, as well as a history of orthopedic wrist surgery.

Significant CRPS requires a comprehensive approach that includes pharmacological agents, physical therapy, psychological support, and, when necessary, interventional pain management procedures [4,5]. Ms. M had a comprehensive treatment regimen that included analgesics, physical therapy modalities, sympathetic nerve blocks, and psychiatric counseling. The goal was to alleviate Ms. M's suffering, strengthen her functional capabilities, and improve her overall quality of life.

Pharmacological agents are critical in the management of CRPS-related pain. Nonsteroidal anti-inflammatory drugs (NSAIDs), gabapentinoids, steroids, opioids, and tricyclic antidepressants may help control neuropathic pain and modulate the abnormal pain signals seen in CRPS [2,5,6]. In Ms. M's case, a tailored treatment regimen was prescribed based on her medical history, concurrent medications, and individual response.

Physical therapy may help CRPS sufferers improve their range of motion, reduce edema, and regain function [7]. To improve limb mobility and relieve pain, several treatments, such as modest mobilization exercises, desensitization methods, mirror therapy, acupuncture, and graded motor imagery, may be used [7,8]. In Ms. M's case, a customized physical therapy program was designed to address her specific impairments and functional limitations.

Sympathetic nerve blocks, such as stellate ganglion blocks or lumbar sympathetic blocks, may be used to relieve pain and restore sympathetic nervous system function in CRPS patients. These treatments work by decreasing the abnormal sympathetic activity that contributes to the duration of CRPS symptoms. Stellate ganglion blocks were used for upper extremity pain, and lumbar sympathetic nerve blocks were administered to Ms. M for lumbar and lower extremity pain. Despite these precautions, her CRPS spread, and Ms. M had additional symptoms throughout her illness, including trigger finger manifestation, trochanteric bursa soreness, and the development of Dupuytren's contracture.

Ms. M's developing CRPS symptoms illustrate the disorder's complexity and multi-system involvement. CRPS is distinguished by swelling, skin abnormalities such as mottling, burning pain, and neurologic dysfunction. These symptoms, together with decreased hand function and limited range of motion, had a significant impact on Ms. M's daily life and led to her deteriorating physical and psychological well-being.

Medication adjustments were made over time to alleviate Ms. M's suffering and improve her quality of life. However, long-term usage of opioids, particularly oxycodone-acetaminophen and fentanyl, should be properly monitored due to potential side effects such as tolerance, reliance, possible withdrawal symptoms from missed doses, and long-term impact on bone health [3,9]. The benefits of steroids such as methylprednisolone in reducing inflammation should be balanced against the risks. Excessive and prolonged use of steroids in such patients may precipitate loss of bone density and uncontrolled hyperglycemia [1,3].

The psychological consequences of CRPS must not be overlooked since they significantly add to the overall burden of illness [4,9,10]. Psychological support and treatment are highly important in managing CRPS. The illness has a significant impact on the patients' mental well-being and quality of life, causing worrisome symptoms like emotional distress, despair, anxiety, and social isolation [10,11]. Ms. M's case exemplifies how the pain caused by CRPS can have a catastrophic impact on mental health, leading to depressive symptoms and anxiety, especially when combined with physical capacity deterioration and the necessity of a cane to help with ambulation. Ms. M's treatment plan included psychological counseling to address the emotional challenges she was experiencing and to teach coping techniques to support her overall well-being. Another highly effective therapy, ketamine, with its antidepressant properties and proclivity to change pain pathways, is an intriguing therapeutic option that was utilized in Ms. M’s treatment plan and addressed both the physical and psychological aspects of CRPS.

It is critical to emphasize that advanced CRPS treatment is often difficult and requires a customized plan based on particular patient needs [11,12]. Because not all patients will respond the same way to the same therapeutic techniques, a trial-and-error approach may be required to find the most beneficial ones [12]. To get the best outcomes, the treatment plan must be evaluated on a regular basis and adjusted as needed.

Avoiding the development of CRPS is critical in the context of orthopedic wrist surgery. Early screening of at-risk individuals, especially women aged 50-70, appropriate surgical methods, and postoperative pain management strategies may all help reduce the development of CRPS [1,2,4]. Furthermore, educating patients about the signs and symptoms of CRPS, promoting early reporting, and providing quick therapy may result in better outcomes for those who do develop the disorder [1,2].

This case highlights the difficulties in diagnosing and managing substantial CRPS after orthopedic wrist surgery. Ms. M's symptoms were treated, and her functional abilities improved using a multimodal strategy that included pharmacologic agents, physical therapy, psychological support, and interventional pain management procedures. Furthermore, this case emphasizes the need for early discovery, precise diagnosis, and comprehensive management for widespread CRPS in order to optimize patient outcomes and improve quality of life.

## Conclusions

An extensive case of CRPS following orthopedic wrist surgery is discussed in this case report and literature review, shedding light on the troublesome character of this condition. The duration of the patient’s symptoms, the spread of CRPS to multiple parts of the body, and the terrible impact on physical and psychological well-being highlight the difficulties of treating CRPS. Several therapies were used to alleviate our patient's pain and improve her functional capabilities during the treatment journey. To address her fluctuating complaints and provide relief, physical therapy, acupuncture, pharmacological treatments such as opioids and adjuvant medicines, interventional pain management procedures, and ketamine infusions were used. However, managing CRPS remains a significant challenge since individual responses to medications might vary, and the potential negative implications of long-term opioid and steroid use must be carefully considered. This case study also emphasizes the need to address the psychological repercussions of CRPS. The degenerative nature of the disease, the loss of function, and the chronic agony that patients like Ms. M suffer may contribute to the development of depression and other psychological disorders. The use of ketamine, with its ability to lower both pain and depression, is a promising avenue for utilization in the treatment of CRPS.
